# The use of implantable cardioverter defibrillators in Iceland: a retrospective population based study

**DOI:** 10.1186/1471-2261-6-22

**Published:** 2006-05-24

**Authors:** Margret Leosdottir, Gudrun Reimarsdottir, Gizur Gottskalksson, Bjarni Torfason, Margret Vigfusdottir, David O Arnar

**Affiliations:** 1 Department of Cardiology, Malmö University Hospital (UMAS), S-205 02 Malmö, Sweden; 2 Department of Medicine, Landspitali University Hospital, Hringbraut, 101 Reykjavik, Iceland; 3 Department of Cardiothoracic Surgery, Landspitali University Hospital, Hringbraut, 101 Reykjavik, Iceland

## Abstract

**Background:**

Indications for implantable cardioverter defibrillator (ICD) implantation have expanded considerably in recent years, resulting in steadily growing numbers of ICD recipients worldwide. The aim of this study was to review the overall experience with ICDs in Iceland.

**Methods:**

This was a retrospective single centre study set at the University Hospital in Iceland. Data on all ICD implantations in Iceland from the first implantation in 1992 till the end of 2002 was reviewed.

**Results:**

Sixty-two patients (71% male) received an ICD during this period. There was an increase in the number of implants by year and the number of new implants in 2001 and 2002 amounted to 56 and 38 per million, respectively. The mean age at implantation was 58 (+/-14) years. Forty patients (65%) had coronary artery disease. The most common indications for ICD implantation were cardiac arrest, 32 (52%) and another 26 (42%) had experienced ventricular tachycardia without cardiac arrest. The most common adverse event was inappropriate shocks. Twenty-eight patients (45%) received therapy from their ICDs, with the majority receiving appropriate therapy. Of the thirteen patients deceased before or during the study period, no case of sudden arrhythmic death was observed.

**Conclusion:**

This study shows that the experience with ICDs in Iceland is in most respects similar to other Western countries.

## Background

The use of implantable cardioverter defibrillators (ICDs) for management of ventricular arrhythmias has become an increasingly popular option since Mirowsky and colleagues first introduced the device in 1980 [1]. Following the publication of several large multi-center trials in the last few years, indications for implantation have expanded considerably and now include prophylactic implantation for patients with coronary artery disease and a severely depressed ejection fraction [2-4]. This has resulted in a rise in implantation rates and thus steadily growing numbers of ICD recipients worldwide, especially in the United States and Western Europe [5].

The objective of this study was to review the experience with ICDs in Iceland where the first ICD implant was performed in 1992. On account of Iceland's small population of approximately 300.000, all of these patients attend the same outpatient clinic at the University Hospital in Reykjavik. Therefore, we were able to review information on every implantation that had been done in the country from the beginning, and as such, examine the experience with ICD therapy in a whole population setting.

## Methods

### Design and subjects

This was a retrospective study, set at the University Hospital in Reykjavik, reviewing all ICD implantations in Iceland from the first implantation in 1992 to the end of year 2002. The study was reviewed and approved by the Institutional Review Committee of Landspitali University Hospital and the Icelandic Data Protection Authority, and complied with the declaration of Helsinki. Informed consent was received from all patients who were alive at the time of the study.

### Data collection and analysis

The following data was collected: Age at implantation; gender; presence or absence of structural heart disease; indication for implantation; ejection fraction (EF) prior to implantation; length of the surgical procedure; type of procedure; surgical complications; adverse events; length of follow up; therapy delivered by the device; malfunctions; reasons for reoperations and causes of death for deceased patients. Information was retrieved from the University Hospital medical records and the ICD outpatient clinic records. Information on EF was retrieved from the latest echocardiogram or ventriculogram performed within one year prior to implantation. When EF was recorded by echocardiography only an estimate was given instead of a single number. For this reason we categorized the EF into four categories: less than 20%, 21–40%, 41–60% and more than 60%. Causes of death were retrieved from the National Death Registry.

When evaluating the cause of delivered therapy by the ICD, stored documentation of shocks and antitachycardia pacing-therapy (ATP) was reviewed. Stored electrograms where available in the the devices implanted in Iceland after1994. As such the majority of ICDs in the patients studied had stored electrograms available. Therapy was categorized as appropriate, inappropriate or indeterminate. Therapy on account of malfunction of the device or documented tachyarrhythmia of atrial origin was defined as inappropriate. Therapy was defined as indeterminate if it could not be categorized as either appropriate or inappropriate. All patients on whom events were evaluated had either stored electrograms or printouts of or event recordings showing a plot of RR intervals during an event. If neither was present the episode was classified as indeterminate with regards to outcome. A single investigator evaluated the stored electrograms and RR plots, with consultation from a second investigator when deemed neccessary.

Therapy was considered effective if the ventricular rate after shock/ATP delivery was <65% of the ventricular rate before shock/ATP delivery. Therapy was classified as causing acceleration of the arrhythmia if ventricular rate was ≥ than 20% faster after shock/ATP delivery than prior to therapy. All ATP given for the same episode of a ventricular tachyarrhythmia was considered a single therapy. Assessment of effectiveness in such episodes was confined to outcome of the last ATP-therapy given. All data are presented as means ± SD.

## Results

### The patients

The demographic and clinical data is shown in Table 1. Sixty-two patients received an ICD in Iceland from the first implantation in April 1992 to the end of December 2002. Prior to the first operation in Iceland one Icelandic patient had received an ICD in Sweden in 1990. He later had generator changes twice in Iceland. Out of these sixty-two patients 44 were male (71%). The mean age at implantation was 58 (+/-14) years (range 16–80 years).

**Table 1:** Patient demographic and clinical data

Age at implantation (years)	Mean	58 +/- 14	
	Range	16–80	
			
Gender	Men	71%	44
	Women	29%	18
			
Underlying cardiac disease	Coronary artery disease (CAD)	62%	40
	Dilated cardiomyopathy	11%	7
	VT/VF without any known cardiac illness	11%	7
	Long QT-syndrome	10%	6
	Hypertrophic cardiomyopathy	2%	1
	Suspected drug related ventricular arrhythmia	2%	1
	Coronary artery spasm resulting in VT	2%	1
	Cardiac amyloidosis	2%	1
			
Indication for implantation	Cardiac arrest due to VT or VF*	52%	32
	VT without loss of consciousness	42%	26
	Syncope, VT/VF induced at EPS*	6%	4
			
EF prior to implantation*	<20%	3%	2
	21–40%	37%	22
	41–60%	20%	12
	>60%	40%	24

* VT = ventricular tachycardia, VF = ventricular fibrillation, EPS = electrophysiologial study, EF = ejection fraction.

Indications for ICD implantations, classified in accordance to AHA/ACC Implantation of Pacemaker and Antiarrhythmia Guidelines, were cardiac arrest due to ventricular fibrillation (VF) or ventricular tachycardia (VT) in thirty-two patients (52%), VT without cardiac arrest in twenty-six patients (42%) and syncope with inducible VT/VF at an electrophysiological study (EPS) in four patients (6%) [2]. No primary prophylactic implantation was performed.

The majority of the patients 40 (65%) had coronary artery disease (CAD). Seven patients (11%) had long QT-syndrome (LQTS) and six (10%) had dilated cardiomyopathy. The following cardiac disease was present in one patient each: hypertrophic cardiomyopathy, coronary artery spasm (refractory to vasodilator therapy and with polymorphic VT) and cardiac amyloidosis. Seven patients (11%) suffered from VT or VF without any demonstrable cardiac illness. Thirty-six patients (60%) had an EF over 40%, including 24 who had an EF >60%. Two deceased patients had no retrievable records of EF.

### The operations

**Figure 1:**
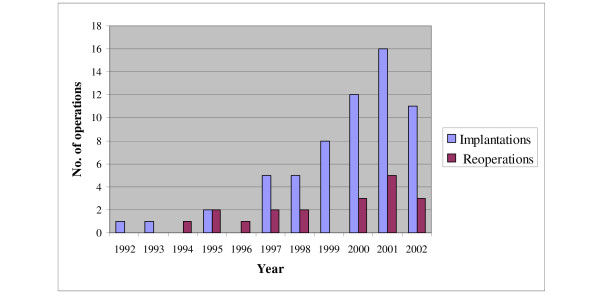
**Yearly number of implantations and reoperations**. Number of implantations and reoperations each year from the first implantation in 1992 till the end of December 2002.

The number of primary implantations and re-operations each year is shown in Figure 1. A total of sixty-one primary implantations and nineteen re-operations were done in the period reviewed. One patient received an ICD in the first year, (1992). Almost two-thirds of the implantations (64%) were performed in the last three years (2000–2002). As can be seen from figure 1 the number of new implants increased each year, although tapering slightly off in the last year. In 2001 and 2002 the number of new implants amounted to 56 and 38 per million, respectively.

The length of the operations decreased during the study period. In the years 1992–1997 the mean operating time were 2:21 hours, compared to 1:20 hours in the years 1998–2002.

All operations were done with a non-thoracotomy approach. Of the sixty-one primary implantations and ten re-operations where a new device was implanted, sixty-four (90%) were implanted subpectorally and seven (10%) under the rectus abdominis muscle. The tranvenous lead insertion was usually via the subclavian vein (89%) but on rare occasions the lead was inserted through the cephalic vein (11%).

Nineteen re-operations were performed involving fourteen patients (Table 2). Twelve out of the nineteen reoperations were related to lead malfunctions; decreased sensing in leads without apparent macroscopic damage at inspection (8), lead insulation damage (2) and minor dislodgement of leads (2). Four elective generator changes were done due to battery depletion. Two reoperations were performed primarily because of inappropriate shocks and one because of loss of connection to the device after shock delivery. No apparent malfunction was demonstrated during detailed lead inspection in any of these three cases.

**Table 2:** Indications for reoperations.

**Related to leads:**	
Decreased sensing in leads	8
Lead or lead insulation breakdown	2
Dislodgement of lead	2
	
**Other:**	
Battery depletion	4
Inappropriate shocks without apparent damage in leads/device	2
Loss of connection to device after shock	1

### Surgical complications and long-term adverse events

These are shown in Table 3. The most common surgical complication was minor bleeding into the device pocket (7). Other surgical complications included serous fluid causing localized swelling in the device pocket (2), pleural effusion (3), and hemothorax (1). In the early postoperative period one superficial skin infection was seen. This was treated with antibiotics and did not result in device removal. Two patients had micro-dislodgement of the leads, and one patient died a week after the ICD implant. The patient suffered from severe congestive heart failure and deteriorated after the operation. His death was not considered to be directly related to the operation, but rather to the severity of his underlying illness.

**Table 3:** Short term (less than 30 days post operatively) and long term (more than 30 days post operatively) adverse events (AE).

**Short term AE **(less than 30 days post operatively):	no. of patients
Haematoma/haemorrhage	7
Seroma	2
Pleural effusion	3
Hemothorax	1
Superficial infection	1
Dislodgement of lead	2
Death	1
	
**Long term AE **(more than 30 days post operatively):	
Inappropriate shocks	
due to atrial fibrillation/flutter	5
due to lead malfunction	2
without apparent cause	3
Decreased sensing/increased pacing threshold	8
Dislodgement of device	1
Discomfort around implant site	1
Loss of connection to device after shock	1

The most common long-term adverse events were inappropriate shocks. In the cases of inappropriate shocks without apparent cause or because of lead malfunction, a new device and/or leads were implanted. Other long-term adverse events were decreased sensing or increased pacing threshold (8), dislodgement of the device (migrated to the axilla) (1), transient discomfort around implant site (1) and in one case there was loss of communication with the device via the programmer wand. All of these cases except the case of discomfort around the implant site resulted in re-operations.

### Therapy

There were 17 dual chamber devices were (24%) and 54 single chamber devices (76%) implanted. All the patients were followed every 3 months at the same device clinic. The mean follow up time was 29.1 +/-18.5 months (range 0.2–154.9 months).

**Figure 2:**
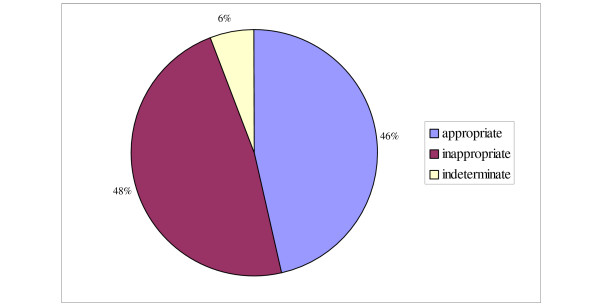
**Classification of shocks**. Therapy was categorized as appropriate, inappropriate or indeterminate. Therapy given to terminate tachyarrhytmia of ventricular origin was defined as appropriate. Therapy on account of malfunction of the device or documented tachyarrhythmia of atrial origin was defined as inappropriate. Therapy was defined as indeterminate if it could not be categorized as either appropriate or inappropriate.

**Figure 3:**
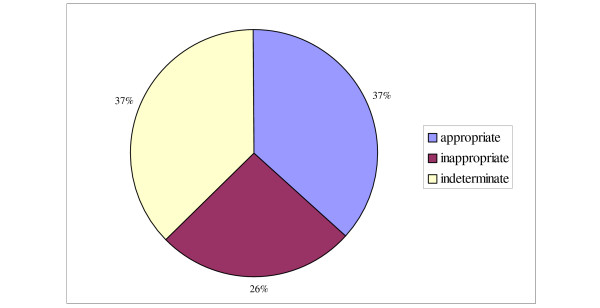
**Classification of ATP-treatments**. Same categorization of appropriateness was used for ATP therapy as for shock therapy (see legend for figure 2).

A summary of shock- and ATP therapy is shown in figures 2 and 3. Twenty-eight patients (45%) received therapy from their ICD during the period studied. Three of the twenty-eight patients were deceased at the time of the data collection and detailed information on therapy for these three patients were unavailable. Thus, a total of 222 shocks and 331 ATP-treatments received by 25 patients were reviewed. The mean number of shocks per patient was 9.7 (range 1–43). The mean number of ATP-therapies per patient was 6.5 (range 1–96).

The majority (84%) of the 25 patients had appropriate therapy at some time from their device. When reviewing effectiveness, 69% of the shocks and 48% of the ATP treatments were effective in reducing the ventricular rate by ≥ 35% which we defined as successful. None of the appropriate shocks caused acceleration of the arrhythmia, but 5% of the appropriate ATP treatments caused acceleration, eventually leading to a shock.

Out of the twenty-five patients receiving treatment, ten (40%) at some point received inappropriate shocks and/or ATP-treatment. The median number of inappropriate shocks was 4 (range 1–33). Four out of these ten patients received inappropriate shocks because of either device or lead malfunction. The remaining six received inappropriate shocks because of atrial arrhythmias. Three of those six patients had dual chamber ICDs. Three patients received the vast majority of inappropriate shocks; 33, 30 and 17 shocks each (83% of the total number of inappropriate shocks). Two received multiple inappropriate shocks because of atrial fibrillation, including one with a dual chamber device. That patient underwent AV-nodal ablation and has not received an inappropriate shock after this. The third patient received 33 shocks on the same day because of device malfunction.

In the majority of cases, inappropriate treatments due to atrial arrhythmias did not result in cardioversion to sinus rhythm. On one occasion, an inappropriate shock for atrial fibrillation resulted in ventricular fibrillation. Three inappropriate ATP-treatments lead to shocks by acceleration of the ventricular rate in atrial fibrillation into the VF zone.

### Survival

Thirteen patients with an ICD died prior to the end of 2002. Eight patients (62%) had a cardiac cause of death; three from acute myocardial infarction resulting in acute heart failure and five from end stage heart failure. No case of sudden arrhythmic death was observed. Two patients died from cancer, two from subarachnoidal haemorrhage and one from bacterial sepsis, unrelated to the device.

## Discussion

In this retrospective study we reviewed sixty-one primary ICD implantations and nineteen re-operations performed in Iceland from the first implantation in April 1992 till the end of December 2002. Major complications and serious adverse events were rare, with the most common long-term adverse event being inappropriate shocks. The majority of patients that received therapy from the device had such appropriately, with 69% of the shocks and 48% of the ATP treatments effectively terminating the arrhythmia.

The main strengths of this study are that it examines the experience with ICD treatment in the Icelandic population as a whole, including every single ICD implantation in the country from the first implantation in 1992 till the end of year 2002. This gives an opportunity to evaluate the effectiveness of ICD therapy in this small community. At the same time the study's major limitation is probably its small sample size and retrospective design.

The age distribution of our patient population was similar to previous studies on this subject [6-9]. The percentage of women was somewhat higher than observed in other studies, or 29% compared to 10–20% [6-9]. Sixty-two percent of our patient population had coronary artery disease as an underlying cardiac illness, which is somewhat lower than observed in other studies [6,7,9-11]. This might be explained by the relatively many cases of long QT syndrome, which is more common in women [10] and usually diagnosed at a younger age, but in our study six patients (10%) had this diagnosis.

The mean EF of our population seemed to be somewhat higher than observed in previous studies [7,8,11]. Forty percent of our patient population had normal ejection fractions and only two patients had an EF lower than 20%.

Distribution of indications according to the AHA/ACC guidelines was similar to previous experiences [6-8,10]. Implantation rates increased rapidly over the ten-year period as was to be expected. When comparing implantation rates in Iceland to other European countries, implants in Iceland account to 56 and 38 per million population in the last two years studied, respectively, which is comparable to the current implant rates of around 45 per million in Western Europe [5,13].

In our study, all of the operations were done by non-thoracotomy approach. No serious infections resulting in device explantation were seen during the study period. Infection rates in ICD patients have been reported to be from 0–8% [14-16]. The fact that all the operations in this study were performed in an operating theatre, as opposed to an electrophysiology laboratory and a rigorous prophylactic antibiotics scheme applied may help explain the low infection rate seen here.

The incidence of haematomas and pleural effusions was somewhat higher than observed in previous studies [6,8,14]. All of the devices were implanted subpectorally or under the rectus abdominis, and this may possibly explain the higher incidence of haematomas and the low infection rate.

The majority of re-operations were performed because of leads problems, most often because of decreased sensing of the lead. In some instances lead malfunction led to inappropriate shocks. Our experience is consistent with previous studies, indicating that lead-related problems account for the large proportion of long term ICD complications [8,15-17].

Inappropriate shocks were the most common long-term adverse event encountered in our study, but 40% (10 out of 25) of the patients receiving shocks, received inappropriate therapy at some time (shocks and/or ATP-therapy). As well as sometimes causing serious physical discomfort for the patient, adverse psychological consequences of multiple shocks can occur. Previous studies have reported worse outcomes on measures of anxiety, depression and quality of life for ICD patients receiving frequent shocks compared to those receiving no or few shocks [19,20]. On the other hand, quality of life in the ICD patient population as a whole has been shown to be comparable to or even superior to medically treated patients with malignant arrhythmias and patients with pacemakers [19,21].

In this study three out of six patients receiving inappropriate therapy because of atrial arrhythmias had dual chamber devices. Studies comparing single chamber devices to dual chamber devices with regard to inappropriate therapy for atrial arrhythmias have shown ambiguous results [22,23]. Currently, a large prospective multicentre randomised trial is underway, designed to analyse the ability of dual chamber ICDs to reduce adverse events, including inappropriate therapy, compared with single chamber devices [24].

Advanced detection algorhythms were activated in the devices during implant in those with known preexisting paroxysmal atrial arrhythmias. These patients all had a dual chamber device implanted. For those who had no prior history of atrial arrhythmias, advanced detection algorhythms were not activated at implant. For those who received inappropriate shocks due to atrial arrhythmias during follow up, advanced detection criteria were activated if the patients had a dual chamber device. Additionally this led to anti-arrhythmic drug therapy if the patient had not been taking such drugs prior to the inappropriate shock.

The vast majority of treated patients received appropriate therapy at some time, with 69% of the shocks and 48% of the ATP treatments being effective in terminating the ventricular arrhythmia. This success rate is somewhat less than previous studies have shown, where as high as 83% success for shock treatment and 78–94% for ATP treatment has been reported [25-28]. However, patient selection, length of follow up, methods of classifying appropriateness and success of therapy vary considerably between studies, making it difficult to reliably compare results.

No death was observed on account of sudden arrhythmic death. While this may indicate the effectiveness of the device to treat life threatening arrhythmias it must also be taken into consideration that that only thirteen patients died during or were deceased before the study period, which may limit the extent to which conclusions can be drawn regarding this.

## Conclusion

In this retrospective study reviewing all ICD implantations in Iceland over a ten year period, we observed that implantation rates have been increasing to a similar extent as in other Western European countries. Therapy received was most often appropriate, with an acceptable success rate in terminating the malignant arrhythmia. Taken together, this study indicates that experience with ICDs in Iceland is comparative to other Western countries, with the devices delivering effective and potentially life-saving therapy to many of the patients. As such, the current study supports the expanding use of ICD therapy for patients suffering from malignant arrhythmias. At the same time we observed that lead problems are not infrequent and inappropriate therapy remains a problem. This underscores the need to focus on improving lead function quality as well as increasing the devices' arrhythmia analysing capability, to minimize the risk of patients receiving inappropriate therapy.

## Competing interests

The author(s) declare that they have no competing interests.

## Authors' contributions

All authors participated in the design of the study. Additionally, ML analysed the data, performed the statistical analysis and drafted the manuscript. GR participated in data collection and analysis. GG and BT revised the manuscript for important intellectual content. MV participated in data collection. DOA participated in data analysis, manuscript drafting and final proofreading/revising. All authors read and approved the final manuscript.

## Pre-publication history

The pre-publication history for this paper can be accessed here:


